# Preventive effects of the novel antimicrobial peptide Nal-P-113 in a rat Periodontitis model by limiting the growth of *Porphyromonas gingivalis* and modulating IL-1β and TNF-α production

**DOI:** 10.1186/s12906-017-1931-9

**Published:** 2017-08-29

**Authors:** Hong-yan Wang, Li Lin, Wei Fu, Hui-Yuan Yu, Ning Yu, Li-si Tan, Jya-wei Cheng, Ya-ping Pan

**Affiliations:** 1 Department of Periodontics and Oral Biology, School of Stomatology, China Medical University, Shenyang, 110002 China; 2 Department of Pharmacy, Tongji Hospital Affiliated with Tongji Medical College, Huazhong University of Science and Technology, Wuhan, 430030 China; 3 Institute of Biotechnology and Department of Medical Science, National Tsing Hua University, Hsinchu, 300 Taiwan; 4 Department of Periodontics and Oral Medicine, University of Michigan, School of Dentistry, Ann Arbor, MI USA

**Keywords:** Antimicrobial peptide, Animal model, Periodontitis, Alveolar bone loss, Inflammatory response, *Porphyromonas gingivalis*

## Abstract

**Background:**

P-113 (AKRHHGYKRKFH-NH2) is a 12-amino-acid histidine-rich peptide derived from histatin 5 that is highly degradable in high salt concentrations and biological fluids such as serum, plasma and saliva. Nal-P-113, a novel antimicrobial peptide whose histidine residues are replaced by the bulky amino acids β-naphthylalanine, causes the antimicrobial peptide to retain its bactericidal activity even in physiological environments. This study evaluated the effect of the novel antimicrobial peptide Nal-P-113 in a rat periodontitis model and the mechanisms of action of Nal-P-113 for suppressing periodontitis.

**Methods:**

Periodontitis was induced in mandibular first molars in rats receiving a ligature and infected with *Porphyromonas gingivalis*. Animals were randomly divided into six groups: a, *P. gingivalis* W83 alone; b, *P. gingivalis* W83 with 6.25 μg/mL of Nal-P-113; c, *P. gingivalis* W83 with 25 μg/mL of Nal-P-113; d, *P. gingivalis* W83 with 100 μg/mL of Nal-P-113; e, *P. gingivalis* W83 with 400 μg/mL of Nal-P-113; and f, control without *P. gingivalis* W83 or Nal-P-113. Morphometric analysis was used to evaluate alveolar bone loss. Microbiological assessment of the presence of *Porphyromonas gingivalis* and total bacteria was performed using absolute quantitative real-time PCR and scanning electron microscopy. Gingival tissue was collected for western blot and immunohistochemical assays of IL-1β and TNF-α levels.

**Results:**

Alveolar bone loss was inhibited by 100 μg/mL or 400 μg/mL of Nal-P-113 compared to the control group (*P* < 0.05). Lower amounts of *P. gingivalis* and total bacteria were found in groups d and e compared with group a (*P* < 0.05). A decrease in the levels of IL-1β and TNF-α was detected in group d and group e compared to the control group (*P* < 0.05). The amount of *P. gingivalis* was positively correlated with IL-1β and TNF-α expression in periodontal tissue (*P* < 0.05).

**Conclusions:**

Nal-P-113 exhibited protective effects on *Porphyromonas gingivalis*-induced periodontitis in rats by limiting the amount of bacteria and modulating IL-1β and TNF-α production. The use of Nal-P-113 in vivo might serve as a beneficial preventive or therapeutic approach for periodontitis.

**Electronic supplementary material:**

The online version of this article (doi:10.1186/s12906-017-1931-9) contains supplementary material, which is available to authorized users.

## Background

Chronic periodontitis is the most common oral disease and the leading cause of tooth loss in adults [1]. Furthermore, it is a known risk factor for many systemic diseases [2–4], such as cardiovascular disease, diabetes, and rheumatoid arthritis.

Periodontitis is primarily initiated by exaggerated host immune responses to periodontal pathogens, which lead to the breakdown of periodontal connective tissues and alveolar bone loss [5]. Among numerous periodontal pathogens, the Gram-negative anaerobic bacterium *Porphyromonas gingivalis* (*P. gingivalis*) is one of the key pathogens in chronic periodontitis. *P. gingivalis* releases copious amounts of virulence factors, including proteases and lipopolysaccharides, which interact with toll-like receptors or penetrate into periodontal tissues and trigger immune responses, resulting in periodontal tissue destruction. Therefore, eliminating periodontal pathogens, particularly *P. gingivalis*, is essential for blocking the process of periodontitis [5]. Local drug therapy is an effective supplementary means for the treatment of periodontitis, for it can reach areas that curettage instruments can’t. Local drug therapies have focused on chlorhexidine and antibiotics such as roxithromycin, spiramycin and metronidazole. Unfortunately, patients have shown poor compliance due to the adverse side effects of these drugs, such as bitter taste, pigmentation, diarrhoea and vomiting. The emergence of drug resistance and dysbacteriosis also limits their clinical application [6, 7]. Therefore, it is urgent to develop novel drugs with few side effects to fight periodontal pathogens. Recently, antimicrobial peptides have become promising antimicrobial agents due to their rapid bactericidal activities and low risk of producing drug-resistant bacterial strains [8]. However, the application of antimicrobial peptides has important drawbacks in vivo due to their high degradation characteristics in the presence of biological fluids [9–12]. Although they demonstrate effective in vitro activities, many antimicrobial peptides have failed in vivo application tests.

P-113 (AKRHHGYKRKFH-NH_2_) is a 12-amino-acid histidine-rich peptide derived from saliva protein histatin 5. Considering its easy degradability, we improved its chemical structure by replacing the histidine residues with bulky amino acid β-naphthylalanine and synthesized a novel antimicrobial peptide, Nal-P-113. This structural alteration enables Nal-P-113 to be resistant to high salt conditions, including PBS, saliva and bovine calf serum [13, 14]. The antimicrobial and anti-biofilm effects of the novel antimicrobial peptide Nal-P-113 have been successfully confirmed in our previous in vitro study together with its nontoxic effect on rat gingival tissue. Nevertheless, to date, no study has focused on the impact of Nal-P-113 in inhibiting periodontitis in an animal model. In this study, we explored the effect of locally administered Nal-P-113 in a rat model of periodontitis by measuring alveolar bone loss, microbe colonization and immune-inflammatory modulation. We postulated that Nal-P-113 can prevent periodontal tissue destruction in experimental periodontitis models not only by controlling the amount of pathogens and total bacteria but also by modulating immune-inflammatory factors in gingiva, representing an auxiliary new approach for periodontitis therapy.

## Methods

### Synthesis, purification, analysis and dilution of antimicrobial peptide Nal-P-113

Nal-P-113, Ac-AKR-Nal-Nal-GYKRKF-Nal-NH_2_, was synthesized from P-113 (AKRHHGYKRKFH-NH2) by replacing His 4, 5 and 12 with β-naphthylalanines based upon the 9-fluorenylmethoxycarbonyl (Fmoc) solid-phase synthesis protocol [13, 15]. Electrospray mass spectrometry was used to identify the peptide, and high-performance liquid chromatography (HPLC) was used to assess its purity. The peptide concentration was determined with a UV spectrophotometer at 280 nm. Nal-P-113 was dissolved in sterile deionized water at concentrations of 6.25 μg/mL, 25 μg/mL, 100 μg/mL and 400 μg/mL.

### Bacteria strains

The Gram-negative bacterium *P. gingivalis* strain W83 was obtained from the American Type Culture Collection (ATCC) and stored at the Department of Oral Biology, China Medical University. *P. gingivalis* strain W83 was cultured at 37 °C for 18 h in an anaerobic chamber with 85% N_2_, 10% H_2_, and 5% CO_2_ in brain heart infusion broth (Difco, Detroit, MI) supplemented with yeast extract (5 mg/mL), haemin (5 μg/mL) and vitamin K_1_ (0.2 μg/mL).

### Ethical statement

The use of rats in this study complied with Animal Research Reporting In Vivo Experiments (ARRIVE) guidelines. The experimental protocols were approved by the ethics committee of China Medical University.

### Animals

Five-week-old, healthy, male Sprague Dawley (SD) rats weighing approximately 200–250 g were used. Rats were randomly assigned to one of the following groups (24 rats for each group): a, *P. gingivalis* W83 alone; *P. gingivalis* W83 with b, 6.25 μg/mL of Nal-P-113; c, 25 μg/mL of Nal-P-113; d, 100 μg/mL of Nal-P-113; e, 400 μg/mL of Nal-P-113; and f, control group without *P. gingivalis* W83 or Nal-P-113.

### Induction of rat periodontitis models

All rats received azithromycin (10 mg/500 mL) for 4 days to reduce the original oral flora before the periodontitis model commenced in a controlled-temperature environment (22 ± 2 °C). This treatment was followed by a 7-day antibiotics-free period. At day 0, rats in all groups (a-f) were anesthetized with 10% chloral hydrate. A 0.2-mm wire was placed in the dentogingival area of both mandibular first molars. Rats in the experimental groups (a-e) received 1 × 10^9^ CFU/mL (1.5 mL) of *P. gingivalis* W83 in the oral cavity and oesophagus when feeding twice per day (at 8:00 a.m. and 8:00 p.m.). Rats in group f, i.e., the control group, received 1.5 mL of BHI broth. Two hours later, Nal-P-113 (1 mL) was dripped into the rats’ periodontal pockets for 1 min in groups b to e. Sterile deionized water was dripped into the periodontal pockets in groups a and f. The above procedures continued for four weeks and were accompanied with inhalational anaesthesia with sevoflurane each time (Hengrui Pharmaceutical Co. Ltd., Shanghai, China).

### Measurement of alveolar bone loss

Four weeks later, all rats (144/144) were healthy. After sacrifice by spinal dislocation, the rat mandibles were separated gently from muscle and soft tissue and exposed overnight to 8% hydrochloric acid. A stereomicroscope (SZX12, Olympus, Japan) equipped with a 12.5× objective was used for observing the morphology of each rat alveolar bone in every group. YC-2008 μ micro-measurement software (Aoka, Suzhou, China) was used for measuring the distance between the enamelo-cemental junction and alveolar bone crest along the axis of the teeth. For evaluating the average alveolar bone loss, three points were measured on the buccal and lingual parts along the axis of the teeth. The average bone loss was calculated for each tooth.

### Bacterial colonization and distribution

Unilateral subgingival plaque and bacteria from the buccal and lingual mucosa were collected using sterile toothpicks and stored in 1× PBS at −80 °C. *P. gingivalis* W83 and total bacteria loads were assessed using absolute quantitative real-time PCR. The primers used were as follows: *P. gingivalis* W83: F: 5′-CATAGATATCACGAGGAACTCCGATT-3′ and R: 5′-AAACTGTTAGCAACTACCGATGTGG-3′. Universal: F: 5′-AACTGGA GGAAGGTGGGGA-3′ and R: 5′-ACGCCAACCTAGTGGAGGA-3′. Each reaction tube contained 20 μL of real-time PCR reaction mixture, including 10 μL of 2× SYBR® Premix Ex Taq^§^, 0.4 μL of 50× ROX Reference Dye II, 0.8 μL of 10 μM primers, 0.4 μL of template DNA and 6.8 μL of ddH_2_O. The amplification cycle conditions were 95 °C for 30 s, 40 cycles of 5 s each at 95 °C, and 34 s at 60 °C. The absolute quantities of the target bacteria were determined using standard curves for *P. gingivalis* W83 and total bacteria. Briefly, the DNA concentrations of the target bacterial strains were serially diluted 10-fold using sterile water to construct standard curves and determine the exact numbers of DNA copies in the samples by calculating ct values. All experiments were performed in triplicate to ensure data accuracy. The data were analysed with ABI 7500 software version 2.0.5 (Applied Biosystems, Foster City, CA, USA) [16].

Contralateral molars were fixed with 2.5% glutaraldehyde (BioChemika, Fluka), washed with PBS and subsequently dehydrated with ethanol. The processed samples were smeared onto a copper plate followed by gold sputtering, and images of the enamelo-cemental junction of the first molar were acquired by scanning electron microscopy (Inspect F50, FEI Company, USA) at 20,000× magnification. Six views were randomly selected from each animal.

### Histopathologic evaluation of periodontal tissue

All mandibular periodontal tissues and tooth samples were divided in half: the left part was immediately cooled to −80 °C for western blotting, and the right part was fixed in 10% formaldehyde solution at 4 °C for one week. The samples were washed with distilled water and placed in 10% EDTA for decalcification for 40 days, rinsed in PBS buffer for 12 h, dehydrated, embedded and cut into 3–4 μm sections for immunohistochemical examination of IL-1β and TNF-α expression in periodontal tissues. Sections were obtained from the tissue that contained tooth, junctional epithelium and gingival tissues. Six views were selected from each sample for mean value calculations. Integrated optical density quantified using a MetaMorph Imaging Microscopic Image Analysis System (UIC, USA) was used to assess the expression of IL-1β and TNF-α. The results were recorded as means ± standard deviations (SD).

### Western blot detection of gingival tissue

Gingival tissue at the buccal root bifurcation was used to examine the levels of IL-1β and TNF-α. A total of 20 mg gingival tissue sample was lysed on ice for 30 min with RIPA lysis buffer containing protease and phosphatase inhibitors (Sigma, St Louis, MO, USA). Supernatant proteins were obtained by centrifugation at 10,000×*g* at 4 °C for 10 min. Total protein was measured with a BCA protein assay kit (Thermo Fisher Scientific, USA). Equal amounts (15 ng) of protein samples were separated by 10% SDS-polyacrylamide gel electrophoresis (PAGE) at 100 V for 2 h and electrotransferred to PVDF membranes (Millipore, Billerica, MA, USA). The membranes were blocked in TBST (20 mM Tris, pH 7.6, 150 mM NaCl, 0.1% Tween 20) containing non-fat dry milk for 2 h at room temperature and incubated overnight in a 1:800 dilution of anti-IL-1β and anti-TNF-α (Kaiji Biological Technology Co. NJ, China) for 2 h. After the membrane was washed three times, it was incubated with a 1:5000 dilution of goat anti-rabbit secondary antibody (Kaiji Biological Technology Co. NJ, China) for 1 h at room temperature and scanned using a fluorescence system with an anti-GAPDH antibody as an internal reference [17]. A gel imaging analysis system (SYNGENE G: BOX ChemiXR5, USA) and Gel-Pro32 software (Media Cybernetic, Inc., USA) were used for densitometry. Statistical analysis was conducted after calculating integral value/internal reference integral value ratios.

### Statistical analysis

All experiments were performed in triplicate for each condition. An ANOVA was used to compare the significance among groups (SPSS Inc., Chicago, IL, USA). Pearson linear correlation analysis was used to calculate the correlation between *P. gingivalis*, total bacteria DNA replication and the expression of IL-1β and TNF-α cytokines. The data are presented as the means ± standard deviation. A *P*-value <0.05 was considered statistically significant.

## Results

### Alveolar bone loss

**Fig. 1:**
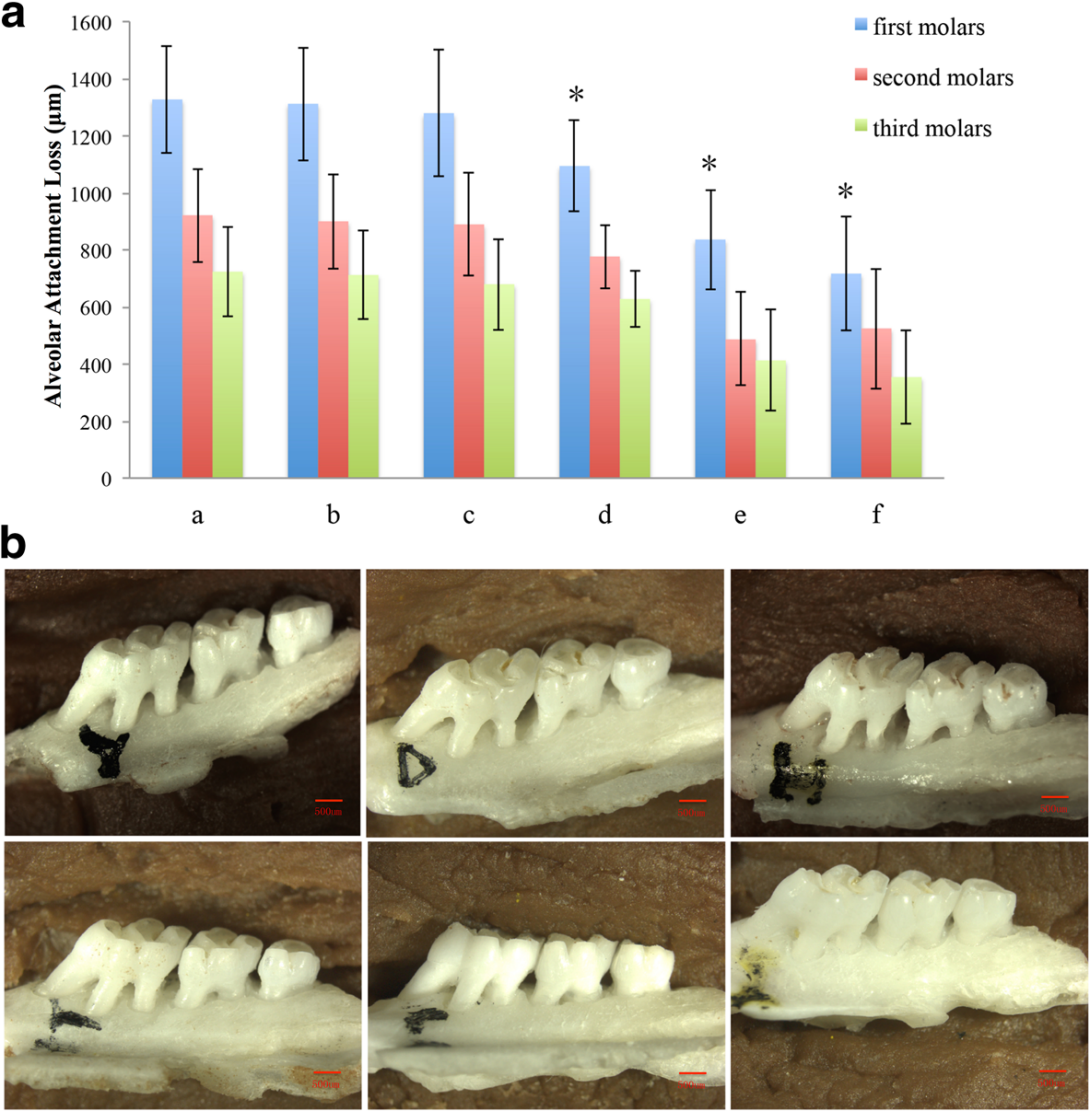
Detection of rat alveolar bone damage by stereomicroscopy and immunohistochemical staining. (**a)** and (**b**) Alveolar bone loss in each group was measured along the long axis of the teeth using a stereomicroscope. **a**: *P. gingivalis* W83 alone; **b**: *P. gingivalis* W83 plus 6.25 μg/mL of Nal-P-113; **c**: *P. gingivalis* W83 plus 25 μg/mL of Nal-P-113; **d**: *P. gingivalis* W83 plus 100 μg/mL of Nal-P-113; **e**: *P. gingivalis* W83 plus 400 μg/mL of Nal-P-113; **f**: control group, without *P. gingivalis* or Nal-P-113. The data are presented as the means of 12 rats in each group (**a**). The images were selected from each group at random (**b**)

First, a rat periodontitis model was successfully established by infecting rats with *P. gingivalis* W83 along with placement of a ligature around the first molars of the rats. Alveolar bone loss is a typical characteristic of periodontitis and is usually used as a marker of periodontitis in this model [18]. A stereomicroscope observation showed that rat alveolar bone was severely damaged in groups a, b and c (Fig. 1B-a, b, c). Administration of Nal-P-113 (100 μg/mL or 400 μg/mL) inhibited the destruction of rat alveolar bone in the mandibular first molar during the course of establishing rat periodontitis models, especially at the location of root bifurcation (Fig. 1B-d, e). Based upon YC-2008 software micro-measurement, the results showed severe connective tissue and alveolar bone destruction characterized by an increased distance between the enamelo-cemental junction and alveolar bone crest in the first molars (1326.40 ± 186.04 μm) in group a (Fig. 1A-a). Nal-P-113 (100 μg/mL or 400 μg/mL) significantly reduced alveolar bone loss in ligated mandibular molars in group d and e compared to that in group a (*P* < 0.05). The average distance (along the axis of the teeth) between the enamelo-cemental junction and the alveolar bone crest in the first mandibular molars in group d and group e was 1094.40 ± 159.04 μm and 836.20 ± 175.03 μm, respectively (*P* < 0.05, Fig. 1A-d and Fig. 1A-e).

### *P. gingivalis* W83 and total bacteria loads

**Fig. 2:**
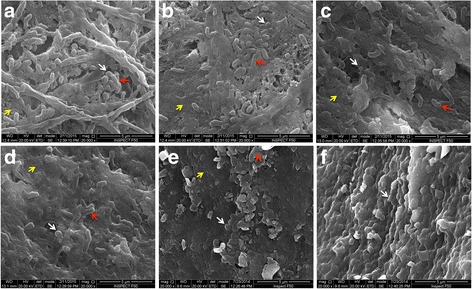
Bacteria on enamelo-cemental junction of rats’ teeth detected by scanning electron microscopy. **a**: *P. gingivalis* W83 alone; **b**: *P. gingivalis* W83 plus 6.25 μg/mL of Nal-P-113; **c**: *P. gingivalis* W83 plus 25 μg/mL of Nal-P-113; **d**: *P. gingivalis* W83 plus 100 μg/mL of Nal-P-113; **e**: *P. gingivalis* W83 plus 400 μg/mL of Nal-P-113; **f**: control group, without *P. gingivalis* W83 or Nal-P-113. SEM magnification, 20,000×. Red arrows indicate *P. gingivalis* W83; white arrows indicate *Coccus*, a genus of resident bacteria in the rat oral cavity; yellow arrows indicate the rat tooth surface

A large amount of bacteria (mainly *Coccus*) present as a biofilm on the surface of teeth could be observed in group f (Fig. 2A-f), which confirmed that the resident flora in the rats’ oral cavity was *Coccus*. Three-dimensional biofilms composed of *Coccus* and *Bacillus brevis* aggregated on the surface of teeth after feeding *P. gingivalis* W83. Administration of Nal-P-113 (100 μg/mL or 400 μg/mL) inhibited dental plaque biofilms in the rat oral cavity, resulting in only few *Coccus* or *Bacillus brevis* colonies scattered on the surface of teeth (Fig. 2A-d and Fig. 2A-e). To further quantify the amounts of bacteria colonizing the rat oral cavity, absolute quantitative real-time PCR was performed to detect *P. gingivalis* W83 and total bacteria. The results illustrated that Nal-P-113 at high concentrations (100 μg/mL or 400 μg/mL) significantly reduced *P. gingivalis* W83 and total bacteria loads (*P* < 0.05, Table 1).

**Table 1:** DNA copy numbers of *P. gingivalis* W83 and total bacteria loads

	group a	group b	group c	group d	group e	group f
*P. gingivalis* W83	(1.74 ± 0.12)E + 17	(1.71 ± 0.10)E + 17	(1.69 ± 0.11)E + 17	(1.06 ± 0.034)E+17^a^	(6.21 ± 0.24)E+16^a^	-
Total bacteria	(2.19 ± 0.29)E + 20	(2.03 ± 0.10)E + 20	(1.98 ± 0.05) E + 20	(1.01 ± 0.051)E+20^a^	(6.97 ± 0.61)E+19^a^	(1.89 ± 0.071)E + 20

^a^ *P* < 0.05, compared with group a

### Expression of IL-1β and TNF-α

**Fig. 3:**
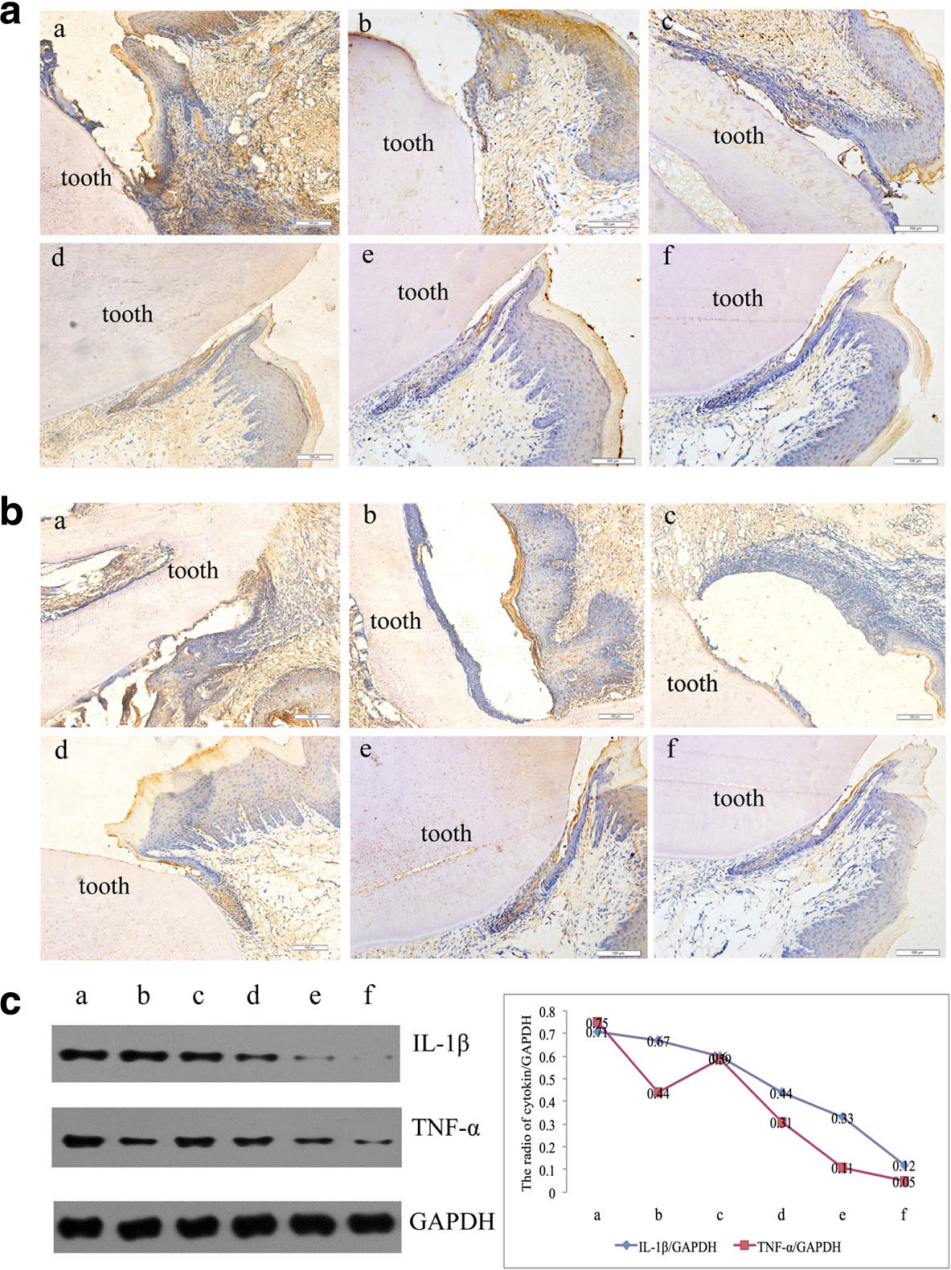
Expression of IL-1β and TNF-α in periodontal tissue. **a** IL-1β expression in the periodontal tissue of rats detected by immunohistochemistry. Brown staining indicates positive immunoreactivity. **b** TNF-α expression in the periodontal tissue of rats detected by immunohistochemistry. Brown staining indicates positive immunoreactivity. **c** Levels of IL-1β and TNF-α detected by western blot analysis. The mean values of IL-1β and TNF-α were measured by densitometry using Gel-Pro32 software. a: *P. gingivalis* W83 alone; b: *P. gingivalis* W83 plus 6.25 μg/mL of Nal-P-113; c: *P. gingivalis* W83 plus 25 μg/mL of Nal-P-113; d: *P. gingivalis* W83 plus 100 μg/mL of Nal-P-113; e: *P. gingivalis* W83 plus 400 μg/mL of Nal-P-113; f: control group, without *P. gingivalis* or Nal-P-113. The data are presented as the means of 12 rats in each group. Magnification, 100×

Immunohistochemical staining was performed, and the percentage of inflammatory cells, including neutrophils, lymphocytes and monocytes, was counted in 6 randomly selected views at 400× magnification. The results showed that in groups a and b, the percentage of inflammatory cells was >75%, accompanied by epithelial spike thickening, periodontal ligament fibre degeneration and alveolar bone damage (Fig. 3A,B-a and Fig. 3A,B-b). In group a, large amounts of IL-1β and TNF-α were found in the periodontal tissue, specifically, the integrated optical density of IL-1β and TNF-α in periodontal tissue was 54.26 ± 2.94 and 27.11 ± 1.60, respectively. Compared to group a, rats in group b and group c demonstrated a slight reduction of IL-1β and TNF-α levels in periodontal tissue (Fig. 3A,B-b and Fig. 3A,B-c), but the differences were not statistically significant (*P* > 0.05, Table 2). With increasing concentrations of Nal-P-113, IL-1β and TNF-α levels in periodontal tissue declined dramatically in group d and group e (*P* < 0.05, Table 2). Additionally, fewer inflammatory cells (< 50%) could be found around the alveolar bone, and the gingival epithelium attached itself closely to the alveolar bone with little periodontal ligament fibre breakage (Fig. 3A,B-d and Fig. 3A,B-e).

**Table 2:** Integrated optical densities of the immunohistochemical staining for IL-1β and TNF-α

	Controls	*P. gingivalis*	Nal-P-113 (*n* = 12)			
	(*n* = 12)	(*n* = 12)	6.25 μg/mL	25 μg/mL	100 μg/mL	400 μg/mL
IL-1β	5.32 ± 0.93	54.26 ± 2.94	49.32 ± 9.81	40.50 ± 5.61	19.78 ± 3.40^a^	12.40 ± 2.51^a^
TNF-α	4.63 ± 0.57	27.11 ± 1.60	24.63 ± 5.17	20.94 ± 3.77	12.62 ± 2.67^a^	9.09 ± 2.07^a^

^a^ *P* < 0.05, compared with the *P. gingivalis* group

Western blot data were consistent with the finding that Nal-P-113 reduced IL-1β and TNF-α expression in gingival tissue in a concentration-dependent manner. In group a, the adjusted values for IL-1β/GAPDH and TNF-α/GAPDH were 0.71 and 0.75, respectively. Four doses of Nal-P-113 all reduced IL-1β and TNF-α levels in gingival tissue compared to those in group a, even though the expression of TNF-α was slightly increased in group c compared with group b (Fig. 3C).

### Correlation between *P. gingivalis* DNA replication and cytokine expression

**Fig. 4:**
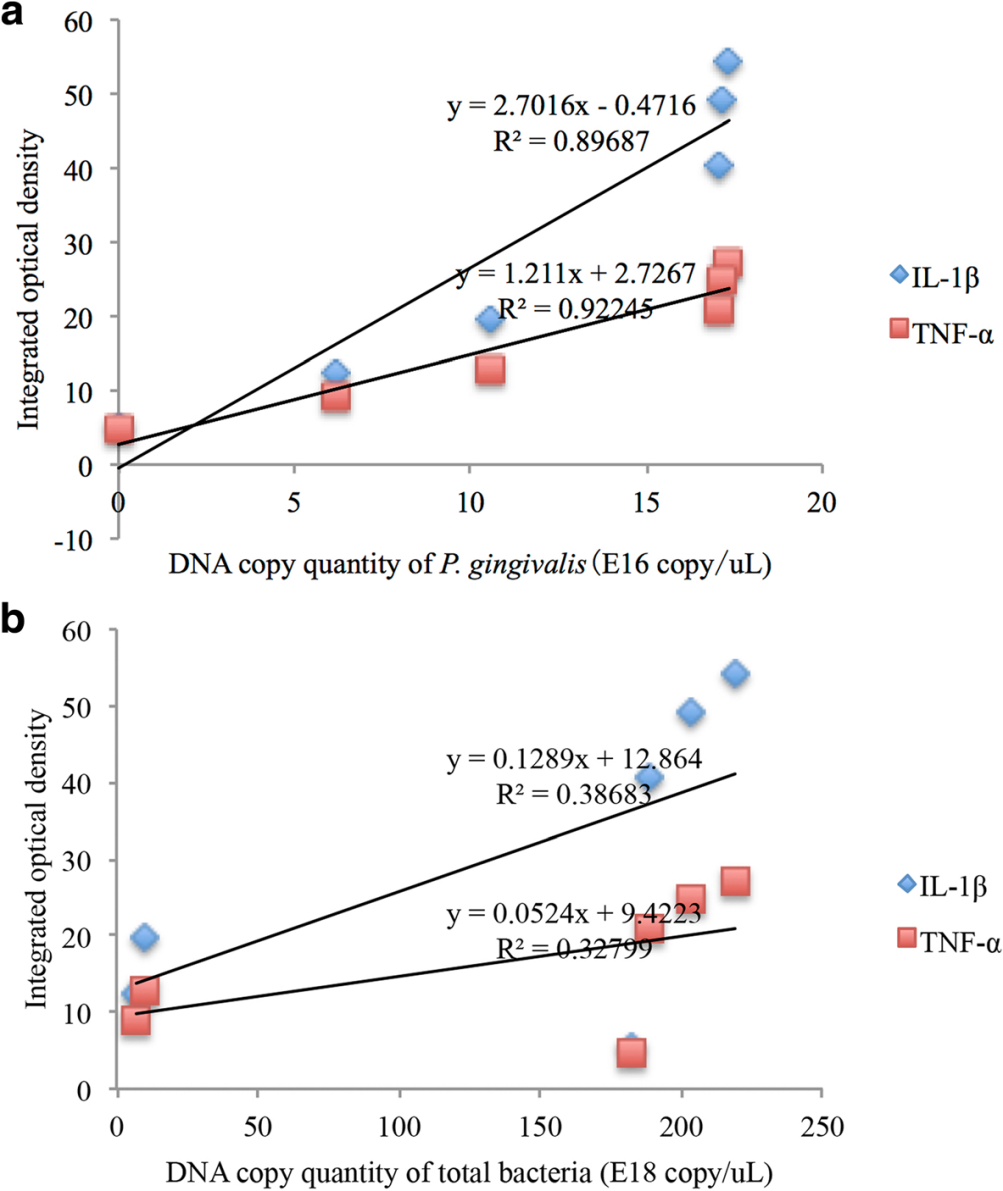
**a** Linear correlation curve of the *P. gingivalis*’ DNA copy number and cytokines in periodontal tissue (IL-1β and TNF-α). The integrated optical densities of IL-1β and TNF-α were calculated on the basis of immunohistochemical staining. The levels of IL-1β and TNF-α in rat periodontal tissue showed a significant positive linear correlation with DNA copy number of *P. gingivalis* (*P* < 0.05). **b** Linear correlation curve of the total bacteria DNA copy number and cytokines in periodontal tissue (IL-1β and TNF-α). The integrated optical densities of IL-1β and TNF-α were calculated on the basis of immunohistochemical staining. There was not a significant positive linear correlation between IL-1β or TNF-α expression levels in rat periodontal tissue and DNA copy number of total bacteria (*P* > 0.05)

A Pearson linear correlation analysis was used to evaluate the correlation between bacterial DNA (*P. gingivalis* and total bacteria) and the expression of IL-1β and TNF-α cytokines. The results showed that both IL-1β and TNF-α expression in periodontal tissue were positively correlated with the copies of *P. gingivalis* DNA (Fig. 4A. IL-1β: *R* = 0.947, *P* = 0.004; TNF-α: *R* = 0.960, *P* = 0.002). Nevertheless, the total bacterial DNA did not correlate with IL-1β or TNF-α expression (Fig. 4B. IL-1β: *R* = 0.622, *P* = 0.187; TNF-α: *R* = 0.573, *P* = 0.235).

## Discussions

Periodontitis is a complex disorder dependent on many factors, but inflammatory cell accumulation induced by microorganisms in periodontal tissues is considered the primary causative factor. *P. gingivalis*, a member of the red complex, is considered one of the most important pathogenic factors of periodontitis, which produces several virulence factors, including lipopolysaccharide, capsules, fimbriae and a group of proteolytic enzymes that damage periodontal tissue [19–21]. In addition, *P. gingivalis* can trigger changes to the amount and composition of the oral commensal microbiota, leading to inflammatory periodontal bone loss [22]. In this condition, the removal of periodontal pathogens, such as *P. gingivalis*, is an effective method to block chronic periodontitis. Although periodontal pathogens are required for disease initiation, the extent and severity of periodontal damage depend on the nature of the host response to bacterial challenge. The development of a novel therapeutic strategy combining both the antibacterial activity and the host-modulatory effect of a new drug could increase the likelihood of successfully managing periodontitis [23]. Some studies have shown that antimicrobial peptides, in addition to exhibiting a direct antimicrobial activity, possess a wide range of immunomodulatory properties [24]. In animal models, researchers have verified that antimicrobial peptides are effective in oral *Candidiasis* infection, lung infection and sinusitis [25–27], but few studies have focused on periodontitis therapy in vivo. The present study evaluated, for the first time, the anti-inflammatory and antimicrobial impact of a novel antimicrobial peptide Nal-P-113 in preventing the progression of periodontitis in a concentration-dependent manner.

In the current investigation, morphometric analysis demonstrated that therapy with Nal-P-113 promoted a significant decrease in bone loss compared to high periodontal breakdown in the non-treated group. The enamelo-cemental junction area served as an observation locus to explore the bacterial distribution on the rat tooth surface. Scanning electron microscopy showed that bacteria adhering to the surfaces of teeth in a scattered distribution were impacted by high concentrations of Nal-P-113. Consistent with the morphological images, quantitative real-time PCR confirmed the results that treatment with 100 μg/mL or 400 μg/mL of Nal-P-113 reduced the amount of *P. gingivalis* and total bacteria loads compared with those in the control group (*P* < 0.05). A previous study showed that Nal-P-113 inhibited bacterial growth even caused bacterial death by permeabilizing and/or forming pores within cytoplasmic membranes in a concentration-dependent manner in vitro [14]. The present study demonstrated the ability of the novel antimicrobial peptide Nal-P-113 to inhibit biofilm formation, local inflammatory response and alveolar bone loss in an experimental rat periodontitis model.

Bacteria are essential for the development of periodontitis but not sufficient to cause disease alone. For periodontitis to develop, a susceptible host is required [28, 29]. Page et al. in 1999 reported that periodontal disease was characterized by high concentrations of matrix metalloproteinases (MMPs), cytokines, and prostaglandins in the periodontal tissue.23 Certain cytokines, including IL-1β and TNF-α, play a critical role in the pathogenesis of periodontitis. IL-1β and TNF-α are both pro-inflammatory cytokines involved in the induction of several other inflammatory mediators in periodontal inflammation, such as IL-6, chemokines, MMPs and prostaglandin E_2_ [30]. Furthermore, IL-1β and TNF-α can induce secondary mediators, resulting in inflammatory response expansion, connective tissue destruction and osteoclastic bone resorption [31]. To explore the host involvement in the disease process, immunohistochemical techniques and western blotting were used to detect IL-1β and TNF-α expression in the periodontal tissue of rats. The results showed that Nal-P-113 reduced IL-1β and TNF-α levels in periodontal tissue in a concentration-dependent manner. Many scholars have also confirmed that other antimicrobial peptides were capable of modulating innate immunity [32–34]. For example, Pingel et al. reported that human beta-defensin (hBD)-3 significantly decreased the secretion of IL-6, IL-10, GM-CSF and TNF-α by human myeloid dendritic cells stimulated with recombinant *Porphyromonas gingivalis* hemagglutinin B [34]. LL-37 induced production of pro-inflammatory cytokines IL-4, IL-5 and IL-1β from human mast cell [35] or by activation of P2X7 receptor from monocytes [36]. Some immune cells, such as macrophages or mast cells, can express G protein-coupled receptors and have a role in both innate immune responses and antimicrobial activation [24]. But in our study, we didn’t find Nal-P-113 alone change IL-1β or TNF-α levels in macrophages (Additional file 1: Figure S1). Other immune cells, such as mast cells, monocytes and neutrophils interact with Nal-P-113 should be investigated in the future studies.

It is worth noting that the TNF-α level detected by western blotting was slightly increased when 25 μg/mL of antimicrobial peptide Nal-P-113 was used. One potential explanation might be that antimicrobial peptides themselves can induce a slight up-regulation of TNF-α expression [37]. Our results showed that 25 μg/mL or 400 μg/mL Nal-P-113 increased TNF-α expression levels in human periodontal ligament stem cells, but not in immortalized human gingival epithelial cells or macrophages (Additional file 1: Figure S1). The specific mechanism is not clear, maybe it decreased the MyD88 and AKT levels to affect the NF-κB signaling pathway and modulated TNF-α response [38]. To some extent, the host-defence mechanisms are sustained by an large network of anti- and pro-inflammatory mediators that may exert antagonistic and/or synergistic biological activities, further studies are required to better understand the role of Nal-P-113 in the modulation of immune-inflammatory responses.

Based on the correlation analysis, IL-1β and TNF-α levels in gingival tissue were positively correlated with *P. gingivalis* but not with the total amount of bacteria. Therefore, in the process of host-microbial interactions, *P. gingivalis* played a more important role in promoting IL-1β and TNF-α expression than other oral bacteria. We surmise that the antimicrobial peptide Nal-P-113 might interrupt pre-inflammatory cytokine expression by reducing *P. gingivalis* loads in the rat oral cavity.

### Conclusions

Overall, the present investigation demonstrates that a novel antimicrobial peptide, Nal-P-113, not only overcomes the degradation susceptibility of antimicrobial peptides in vivo but also exerts potent preventive effects on rat periodontitis. This peptide inhibits *P. gingivalis*-induced local inflammatory response and alveolar bone loss by reducing the amount of bacteria directly and down-regulating pro-inflammatory molecules such as IL-1β and TNF-α. Though further studies are needed to determine the exact mechanism of host-microbial interactions in periodontitis treated with antimicrobial peptides, these agents have the potential to be used as a new preventive or therapeutic strategy for chronic periodontitis in the clinic.

## Additional file

Additional file 1: Figure S1. (A) IL-1β levels of immortalized human gingival epithelial cells, human periodontal ligament stem cells and macrophages after co-cultured with Nal-P-113 for 2 h. Nal-P-113 (25 μg/mL or 400 μg/mL) did not change the levels of IL-1β expression in immortalized human gingival epithelial cells, human periodontal ligament stem cells or macrophages detected by enzyme-linked immunosorbent assay (*P* > 0.05). (B) TNF-α levels of immortalized human gingival epithelial cells, human periodontal ligament stem cells and macrophages after co-cultured with Nal-P-113 for 2 h. Nal-P-113 (25 μg/mL or 400 μg/mL) increased TNF-α levels in periodontal ligament cells (*P* < 0.05), but not in gingival epithelial cells or macrophages (*P* > 0.05). (JPEG 453 kb)

## Abbreviations

CFU: Colony forming units
HPLC: High-performance liquid chromatography
IL-1β: interleukin-1 beta
MMPs: Matrix metalloproteinases
qPCR: Quantitative PCR
SD rats: Sprague Dawley rats
SEM: Scanning electron microscopy
TNF-α: tumour necrosis factor-alpha.
