# Supplementary figures and images for: Preventive effects of the novel antimicrobial peptide Nal-P-113 in a rat Periodontitis model by limiting the growth of Porphyromonas gingivalis and modulating IL-1β and TNF-α production
Source: BMC Complement Altern Med. 2017 Aug 29;17:426. doi: 10.1186/s12906-017-1931-9 (PMC5576277; doi:10.1186/s12906-017-1931-9)

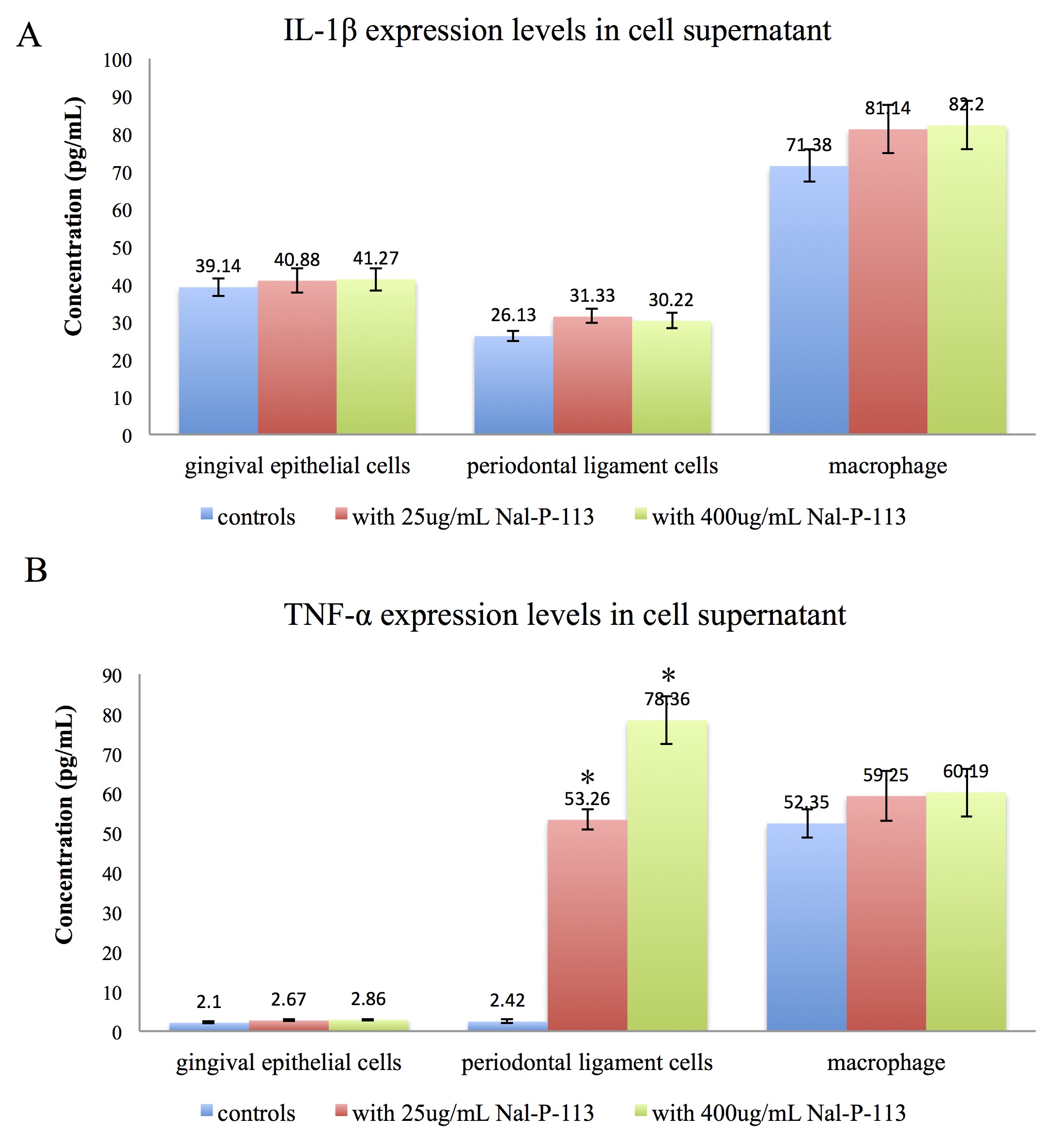

Supplement: Additional file 1: Figure S1. — (A) IL-1β levels of immortalized human gingival epithelial cells, human periodontal ligament stem cells and macrophages after co-cultured with Nal-P-113 for 2 h. Nal-P-113 (25 μg/mL or 400 μg/mL) did not change the levels of IL-1β expression in immortalized human gingival epithelial cells, human periodontal ligament stem cells or macrophages detected by enzyme-linked immunosorbent assay (P > 0.05). (B) TNF-α levels of immortalized human gingival epithelial cells, human periodontal ligament stem cells and macrophages after co-cultured with Nal-P-113 for 2 h. Nal-P-113 (25 μg/mL or 400 μg/mL) increased TNF-α levels in periodontal ligament cells (P < 0.05), but not in gingival epithelial cells or macrophages (P > 0.05). (JPEG 453 kb) [file 12906_2017_1931_MOESM1_ESM.jpg]
